# Design and Evaluation of a Bilateral Semi-Rigid Exoskeleton to Assist Hip Motion

**DOI:** 10.3390/biomimetics9040211

**Published:** 2024-03-30

**Authors:** Arash Mohammadzadeh Gonabadi, Prokopios Antonellis, Alex C. Dzewaltowski, Sara A. Myers, Iraklis I. Pipinos, Philippe Malcolm

**Affiliations:** 1 Department of Biomechanics, and Center for Research in Human Movement Variability, University of Nebraska at Omaha, Omaha, NE 68182, USA; antonelp@ohsu.edu (P.A.); adzewaltowski@unomaha.edu (A.C.D.); samyers@unomaha.edu (S.A.M.); pmalcolm@unomaha.edu (P.M.); 2 Institute for Rehabilitation Science and Engineering, Madonna Rehabilitation Hospitals, Lincoln, NE 68506, USA; 3 Department of Neurology, Oregon Health & Science University, Portland, OR 97239, USA; 4 Scholl College of Podiatric Medicine, Rosalind Franklin University of Medicine & Science, North Chicago, IL 60064, USA; 5 Department of Surgery and Research Service, Nebraska-Western Iowa Veterans Affairs Medical Center, Omaha, NE 68105, USA; ipipinos@unmc.edu; 6 Department of Surgery, University of Nebraska Medical Center, Omaha, NE 68105, USA

**Keywords:** exosuit, exoskeleton, force-tracking, metabolic cost, actuation magnitude, timing, walking, biomechanics, robotics, biomedical engineering

## Abstract

This study focused on designing and evaluating a bilateral semi-rigid hip exoskeleton. The exoskeleton assisted the hip joint, capitalizing on its proximity to the body’s center of mass. Unlike its rigid counterparts, the semi-rigid design permitted greater freedom of movement. A temporal force-tracking controller allowed us to prescribe torque profiles during walking. We ensured high accuracy by tuning control parameters and series elasticity. The evaluation involved experiments with ten participants across ten force profile conditions with different end-timings and peak magnitudes. Our findings revealed a trend of greater reductions in metabolic cost with assistance provided at later timings in stride and at greater magnitudes. Compared to walking with the exoskeleton powered off, the largest reduction in metabolic cost was 9.1%. This was achieved when providing assistance using an end-timing at 44.6% of the stride cycle and a peak magnitude of 0.11 Nm kg^−1^. None of the tested conditions reduced the metabolic cost compared to walking without the exoskeleton, highlighting the necessity for further enhancements, such as a lighter and more form-fitting design. The optimal end-timing aligns with findings from other soft hip exosuit devices, indicating a comparable interaction with this prototype to that observed in entirely soft exosuit prototypes.

## 1. Introduction

While the development of exoskeletons that target metabolic cost reduction has focused on aiding the ankle joint [1,2], there is potential for greater reductions by assisting other joints. Research has shown that the hip joint contributes up to 45% of the mechanical power during walking [3], leading to a growing interest in extending assistance to this area [4,5,6]. The distinctive muscle characteristics and reduced prominence of efficient elastic tendons in hip joint muscles could cause the hip to consume more energy than the ankle, which has more efficient energy storage and return from the Achilles tendon [3,7,8]. Despite this, reductions achieved by assisting the hip joint still appear to be lower than those achieved with ankle exoskeletons [9,10,11,12,13]. More research is needed to better understand the relationships between energetic benefits and assistance levels at the hip [6,14,15,16].

Assisting at the hip has the added advantage of positioning the device closer to the body’s center of mass, thereby minimizing the added mass penalty [17]. Experimental studies and simulations also reveal that exoskeletons can assist muscles from multiple joints, even those they do not directly cover [18]. For example, an ankle exoskeleton can indirectly assist the hip [19]. This capability offers an exciting opportunity to combine the advantages of minimizing the mass penalty and assisting at the hip. Such a combination can be particularly beneficial in patient populations prone to ulcer formation at the feet. Approximately 40% of the metabolic cost during level walking is attributed to the hip muscles [7,20,21]. Thus, various groups have developed rigid exoskeletons and soft exosuits to assist the hip [4,6,8,22,23]. Soft exosuits, in particular, allow for greater freedom of movement [6]. However, they often cannot apply the same torque magnitudes as rigid exoskeletons and must rely on skin friction to remain anchored [22].

Both rigid and soft exoskeletons present merits and challenges. While rigid exoskeletons enable the generation of controllable and accurate assistive torques, they are more complex and heavier due to misalignment issues [24]. In contrast, soft exoskeletons can address this misalignment problem because of their elasticity yet still require skin friction for anchoring, potentially causing discomfort [24]. Recently, there has been a shift toward developing semi-rigid exoskeletons that merge the beneficial characteristics of both rigid and soft exoskeletons. Zhang et al. [24] introduced a semi-rigid knee exoskeleton to minimize misalignment while retaining some aspects of both designs. Schmidt et al. [25] developed a Myosuit to provide continuous assistance at the hip and knee joints when working with and against gravity while performing daily activities. Lin et al. [26] also accomplished a compliant and accurate model in their innovative soft–rigid hand exoskeleton. Developing semi-rigid exoskeletons to become more mechanically efficient is an essential topic of ongoing research.

Here, we detail the development of a bilateral semi-rigid hip exoskeleton end-effector to assist hip extension during walking. Our semi-rigid design allows for more freedom of movement than a traditional hinged hip exoskeleton. Our study aimed to optimize the mechanical components and controller settings to maximize force-tracking performance. We hypothesized that a specific series of elastic stiffness and controller gain settings would maximize accuracy. Our study also investigated the effect of one assistance timing and one assistance magnitude parameter on metabolic cost during walking. We hypothesized that (1) the timing of peak assistance would affect metabolic cost following a U-shaped trend versus timing, and (2) increasing magnitudes of assistance would lead to a monotonic reduction in the metabolic cost of walking.

## 2. Materials and Methods

### 2.1. Hip Exoskeleton Design

**Figure 1:**
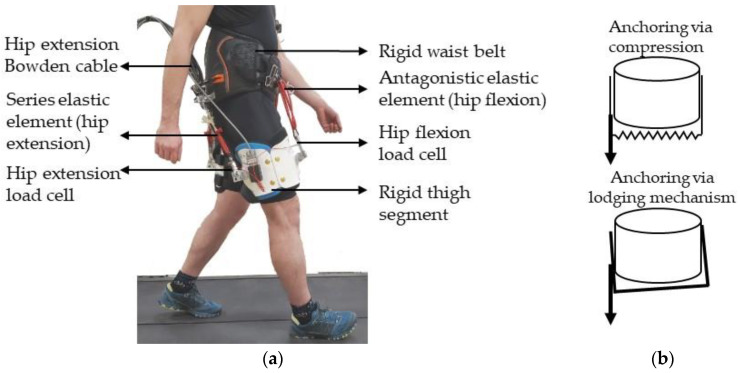
Exoskeleton Design. (**a**) Components of the semi-rigid hip exoskeleton. (**b**) Anchoring mechanism of the semi-rigid exoskeleton. The exoskeleton anchors to the waist through a combination of compression forces and a mechanism in which the waist belt becomes lodged when the actuation force attempts to rotate the waist belt.

We developed a semi-rigid hip exoskeleton end-effector intended to allow more freedom of movement than a hinged hip exoskeleton while improving anchoring to the body segments (Figure 1, Video). The waist belt and thigh segments are rigid but not connected via hinges. The hip exoskeleton is linked to a commercially available, high-powered, off-board actuation system (HuMoTech, Pittsburgh, PA, USA), applying forces to the dorsal side of the hip to assist in hip extension. On the frontal side of the hip, a set of springs provides passive hip flexion assistance. Safety features include a remote stop button, a software force limit (software fuse) to stop the motor if load cell force exceeds 300 N, and a mechanical fuse consisting of a thin piece of rope that disconnects if the tension exceeds its breaking strength.

### 2.2. Exoskeleton Controller

**Figure 2:**
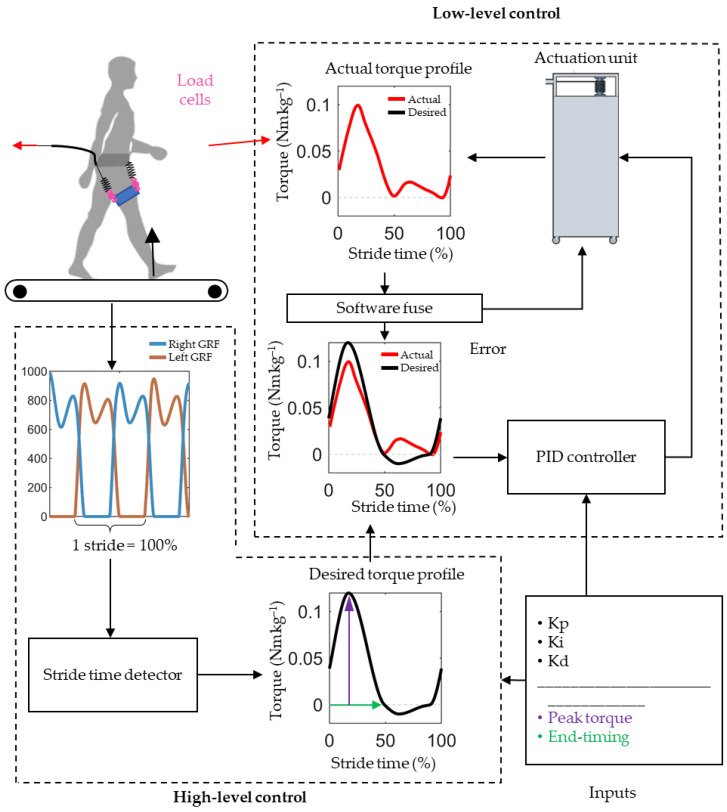
Control algorithm overview: This figure illustrates the high-level control strategy, where the controller sets desired torque (black) profiles based on stride time. The profiles indicate the target torque that the controller aims to provide, not the hip joint’s actual output. A low-level PID controller is then employed to adjust the motor velocity, ensuring precise adherence of the actual torque (red) measured by the load cell to these target profiles throughout the stride.

We developed a high-level temporal controller that allows the independent application of sinusoidal extension and flexion torque profiles to each leg as a function of the stride cycle percentage (Figure 2). We measured the ground reaction forces of both legs at 1000 frames per second using a split-belt force treadmill (Bertec, Columbus, OH, USA) and estimated the percentage of stride time based on the most recent heel contact time and a moving average of previous steps. The controller calculated the forces needed to achieve a specific net torque during hip extension, given that the forces were generated by the hip flexion springs and that the torque arm was assumed to be 10 cm. Given the absence of specific data regarding the moment arm in the context of our study, we made an assumption based on typical anatomical dimensions [27]. The choice of a 10 cm moment arm length was selected as an approximation, taking into account average body proportions [27]. However, this assumption may introduce limitations regarding accurate moment measurement and control. For future studies, employing more precise measurement techniques such as sensors or markers to directly assess the moment arm could enhance the accuracy of our biomechanical analyses. The error between the actual forces, measured with the load cell, and the desired forces was minimized using a low-level controller developed by HuMoTech, which employs a closed-loop proportional–integral–derivative (PID) algorithm with configurable gains [28]. A control station comprising an input–output interface (HuMoTech) and a real-time computer (SpeedGoat, Liebefeld, Switzerland) runs the controller in Simulink (MathWorks, Boston, MA, USA).

Our control strategy, including the implementation of the PID controller, follows the methodology described in our previous work [29]. This approach utilizes a temporal force-tracking control law (Equation (1)), where the desired torque profiles are adjusted based on the stride cycle. The control hardware operates at a sampling rate of 1000 Hz, optimized for real-time feedback, ensuring accurate force tracking.

(1) $$u(t)=K_{p}\times e(t)+K_{i}\times \int_{0}^{T}e(\tau )\;d\tau +K_{d}\times \frac{de(t)}{dt}$$

The provided equation represents the output *u*(*t*) of a PID controller, which adjusts system input based on three terms—proportional (*K_p_*), integral (*K_i_*), and derivative (*K_d_*)—to maintain the desired output level. The proportional gain (*K_p_*) multiplies the current error (*e*(*t*)), which is the difference between the desired setpoint and the actual process variable, aiming to reduce the error. The integral gain (*K_i_*) integrates the error over time to eliminate residual steady-state error, and the derivative gain (*K_d_*) predicts future errors based on the current rate of change, allowing the controller to take preemptive actions. These components form the control signal, *u*(*t*), which drives the system to minimize the error, achieving stability and desired performance through proper tuning of the *K_p_*, *K_i_*, *K_d_*, and constants.

We selected the general shape of the actuation profile based on a review of force profiles in the literature [4,6,8,22,23]. Considering the limits of the forces we could achieve, we chose to model the force profile after the shape of the one used by Lee et al. [4], which resulted in a substantial 21% reduction in metabolic cost with a relatively low peak moment of around 7.5 Nm. Inspired by the optimal torque profile in that study, we set the desired onset of the extension torque at 90% of the stride, with a peak extension torque timing at 17% of the stride cycle. Under different conditions, we varied the end timing of the extension torque and peak magnitude.

### 2.3. Exoskeleton Design and Control Optimization Protocol

We conducted several single-participant analyses, including optimization of the stiffness of the series elastic element of the hip extension actuation and the gains of the PID controller to determine the optimal device and control parameters. Torque tracking was assessed by calculating the root-mean-square error (RMSE) between the actual and desired torque. We measured how well the system tracked the torque profiles within each stride by calculating the RMSE for each stride’s actual and desired torque time series. Additionally, we reported the RMSE of the average torque per step to gauge how well the system tracked the average torque per stride [29,30]. We employed an oscillation-level metric to detect unwanted high-frequency oscillations [31]. This metric is obtained by high-pass filtering the error with a 10 Hz cut-off frequency and then integrating the energy spectral density. All analyses were performed in MATLAB (MathWorks).

### 2.4. Metabolic Cost and Biomechanics Evaluation Protocol

**Figure 3:**
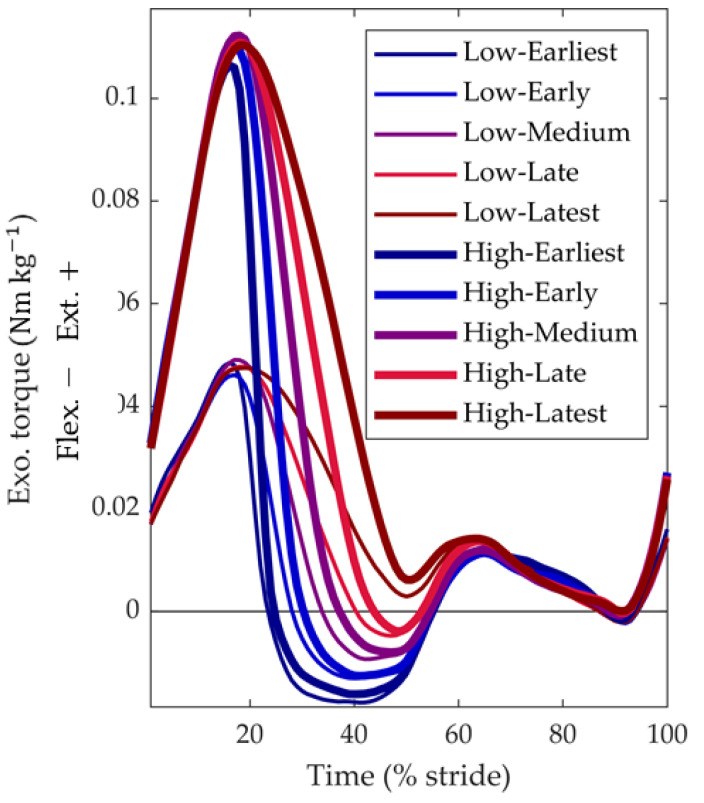
Actuation profiles: the 10 actuation profiles were used in the metabolic cost and biomechanics evaluation protocol.

Ten healthy adults (four males, six females; age: 27.6 ± 5.9 years, body mass: 65.3 ± 13.1 kg, height: 1.66 ± 0.08 m) participated in this study. The testing session started with a five-minute standing trial to measure the resting metabolic rate, followed by a warm-up of approximately 20 min. During this warm-up, we cycled through all the force profile conditions and adjusted the gain-tuning settings for individuals if needed. During the testing protocol, we maintained the onset and peak of the extension torque constant while varying the end-timing and peak magnitude across 10 conditions. These conditions included combinations of five desired end-timings, ranging from 21% to 49%, and two desired peak torque magnitudes, ranging from 0.06 to 0.12 Nm kg^−1^ (Figure 3). In addition to these ten conditions, participants walked in two reference conditions: one without actuation (Powered-Off) and another without wearing the exoskeleton (No-Exo). The entire protocol was split into three blocks in which conditions were completed back-to-back. Each block had low and high magnitudes and different timings presented in random order. The first and last condition of each block lasted five minutes. All the other trials lasted two minutes. Between the blocks, participants rested for at least 10 min.

We measured oxygen consumption and carbon dioxide production using indirect calorimetry (K5, Cosmed, Rome, Italy). By applying the Brockway equation [32], we converted the breath-by-breath measurements to watts per kilogram (W kg^−1^). We averaged the breath-by-breath data in the final two minutes to estimate the steady-state metabolic rate of the resting trial and the conditions at the beginning of each block. We estimated the steady-state metabolic rates for conditions lasting only two minutes by fitting the breath-by-breath data immediately after transitioning to each new condition until just before the change to the following condition with an exponential function and estimating the asymptote [33,34]. We calculated the net metabolic rate of walking by subtracting the metabolic rate of standing at rest from the metabolic rate under each walking condition.

We recorded 3D kinematics using motion capture (VICON Vero, Oxford Metrics, Yarnton, UK; 2000 Hz) from 23 reflective markers placed on anatomical landmarks on the skin, tight-fitting suit, or exterior of the shoes according to a modified Helen Hayes marker set [35]. Kinematic data were tracked from each condition in Nexus software version 2.12 (Vicon Motion Systems Inc., Oxford, UK) and exported as a Visual 3D file (Visual 3D, C-Motion, Maryland, MD, USA) for further processing. We filtered the ground reaction forces with a low-pass Butterworth filter with a 6 Hz cut-off and processed motion capture data using OpenSim (version 4.0, SimTK, San Jose, CA, USA; [36,37]) with the model from Rajagopal et al. [36,38]. After scaling the model based on motion capture data from a static pose with the ‘adjust model markers’ and ‘preserve mass distribution’ options, we adjusted the calculated mass distribution to account for the exoskeleton’s mass (5.77 kg). We used the inverse kinematics tool to estimate joint kinematics from the marker data, restricting the ankle and knee motion to one degree of freedom (flexion and extension) but utilizing all three degrees of freedom for the hip to simulate walking’s hip movement realistically. We applied the inverse dynamics tool with a 6 Hz cut-off filter setting for marker coordinates to compute joint moments.

To calculate the exoskeleton’s applied torque on the hip joint, we measured the force of each sensor on the exoskeleton and multiplied this by the lever arm versus the hip. We assumed a fixed value (10 cm) for the lever arm. The multiplication of the force and lever arm yielded the applied torque on the hip joint. To calculate the biological moment for the hip, we subtracted the exoskeletal torque from the total moment. For the ankle and knee, we report the total external moments. In addition to investigating the effects on the hip, knee, and ankle moments and powers, we calculated individual leg power using the methods described in Donelan and Kuo [39]. Firstly, we computed the center-of-velocity relative to the reference frame attached to the treadmill belt by dividing the ground reaction force measured by the treadmill by body mass, integrating over time, and adjusting for treadmill velocity. Subsequently, we determined the right leg’s power by multiplying the right leg’s ground reaction force by the center-of-mass velocity. Finally, we computed the work rate during characteristic negative and positive work phases in the leg power: collision, rebound, preload, and push-off.

Finally, we analyzed the processed data in MATLAB (MathWorks). We segmented all data into strides that began at the ipsilateral heel strike and ended with the next ipsilateral heel strike based on foot contact detection from ground reaction force data. Outliers were detected by examining how well signals remained within a band defined by the median ± 1.5 times the interquartile range and removed outlying strides [40].

### 2.5. Statistical Analyses

All metabolic cost and biomechanical variables were analyzed by reporting means and standard error across participants for each condition. We conducted a linear mixed-effects model analysis to examine the effects and interactions of the timing and magnitude of torque profiles on the metabolic rate and biomechanical variables (Equation (2)). In this analysis, the participants were considered to be the random factor. Since there were only two magnitude settings, we evaluated only the linear effect of magnitude in the equation. We considered a second-order effect for timing, hypothesizing a U-shaped trend in metabolic cost versus timing. We multiplied both timing terms by magnitude because the effect of timing could not exist without actuation magnitude. Additionally, we included a constant offset term (*c_4_*), resulting in the following statistical model:

*Y* ≈ *τ_exo_* × (*c*_1_ × *t^2^_exo_* + *c*_2_ × *t_ex__o_*) + *c*_3_ × *τ_exo_* + *c*_4_ (2)

In this equation, *Y* is the metabolic or biomechanical variable that is being analyzed, and *t_exo_* and *τ_exo_* represent the desired end-timing (% gait cycle) and peak torque magnitude (Nm kg^−1^), respectively. *c*_1_ and *c*_2_ are coefficients that quantify the second-order and linear effects of timing (*t_exo_*) on the assisted hip extension moment, respectively. These coefficients were determined through mixed-effects model analysis, reflecting the curvature (for *c*_1_) and slope (for *c*_2_) of the relationship between timing and the biomechanical effect of the exoskeleton’s assistance. We started with this initial model and removed non-significant terms stepwise until only significant terms remained. To compare the differences between the Powered-On conditions versus the Powered-Off and No-Exo conditions, we conducted paired *t*-tests. We performed all statistical analyses in MATLAB (MathWorks) and set the significance threshold at 0.05.

## 3. Results

### 3.1. Device Optimization Results

Series elasticity is known to influence actuator control performance, with high stiffness offering quicker response times but possibly more noise and low stiffness, potentially enhancing force tracking but causing a delay [31,41]. To find the optimal stiffness for the spring in series with the hip extension actuation, we tested nine different springs ranging from 892 to 30,000 Nm^−1^. We examined the effects on within-stride RMSE, between-stride RMSE, and oscillation level using effective stiffness measured by plotting force change against length change as an independent parameter. The results did not reveal a clear single trend, possibly due to varying spring designs from different suppliers. Nevertheless, a stiffness of 4438 Nm^−1^ was generally found to best balance the difference error metrics, and this value was used in subsequent tests.

**Figure 4:**
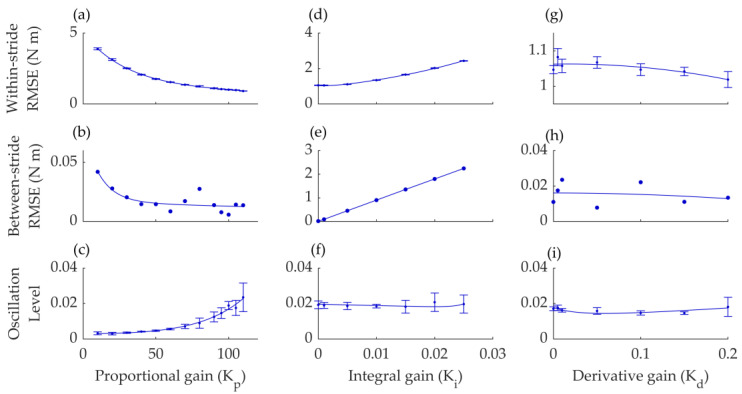
Gain tuning results: (**a**–**c**) Effects of tuning proportional gain (*K_p_*) on within-stride RSME, between-stride RMSE, and oscillation level. (**d**–**f**) Effects of tuning integral gain (*K_i_*) on within-stride RSME, between-stride RMSE, and oscillation level. (**g**–**i**) Effects of tuning integral gain (*K_d_*) on within-stride RSME, between-stride RMSE, and oscillation level. Based on these tests, in further experiments, *K_p_*, *K_i_*, and *K_d_* were set to 95, 0, and 0, respectively. Error bars in all panels represent standard deviation between strides, indicating variability in the tuning results (*n* ≈ 19, 12, and 9 strides per condition for *K_p_*, *K_i_*, and *K_d_* sweep). The middle row (plots **b**,**e**,**h**) does not have error bars since there is only one between-stride RMSE value per gain setting.

Next, we performed a series of experiments to investigate the optimal settings for the three force-tracking parameters: the proportional gain (*K_p_*), which modifies the actuator control signal relative to the error; the integral gain (*K_i_*), which adjusts the control signal based on the accumulated integral of the error; and the derivative gain (*K_d_*), which adjusts the control signal based on the derivative of the error. First, we varied *K_p_* from 10 to 110, holding *K_i_* and *K_d_* at 0. Based on this experiment, we identified the optimal *K_p_* to be 95 (Figure 4a–c). Next, we changed *K_i_* from 0 to 0.025, keeping *K_p_* at its optimized setting and *K_d_* at 0. Based on this second sweep, we identified the optimal *K_i_* at 0 (Figure 4d–f). Finally, we evaluated the effect of changing *K_d_* from 0 to 0.2 while keeping *K_p_* at 95 and *K_i_* at 0 (Figure 4g–i). This test did not show a clear effect of *K_d_*; hence, we kept *K_d_* at 0 for future tests. *K_d_* and *K_i_* did not have a clear effect, possibly because the optimized spring improves force tracking.

The optimized settings produced a within-stride RMSE of 1.06 Nm, a between-stride RMSE of 0.008 Nm, and an oscillation level of 0.014. In the further metabolic cost and biomechanical parameter evaluations, we only slightly adjusted *K_p_* depending on the participant but kept *K_i_* and *K_d_* at 0.

### 3.2. Metabolic Cost and Biomechanics Evaluation Protocol Results

The programmed desired actuation profile conditions resulted in actual end-timings ranging from 24.8 ± 1.3% of the stride (mean ± S.D. of all early conditions) in the earliest condition to 49.6 ± 1.1% in the latest condition. The peak magnitudes ranged from 0.0479 ± 0.0084 Nm kg^−1^ in the low magnitude conditions to 0.1102 ± 0.0163 Nm kg^−1^ in the high magnitude conditions.

**Figure 5:**
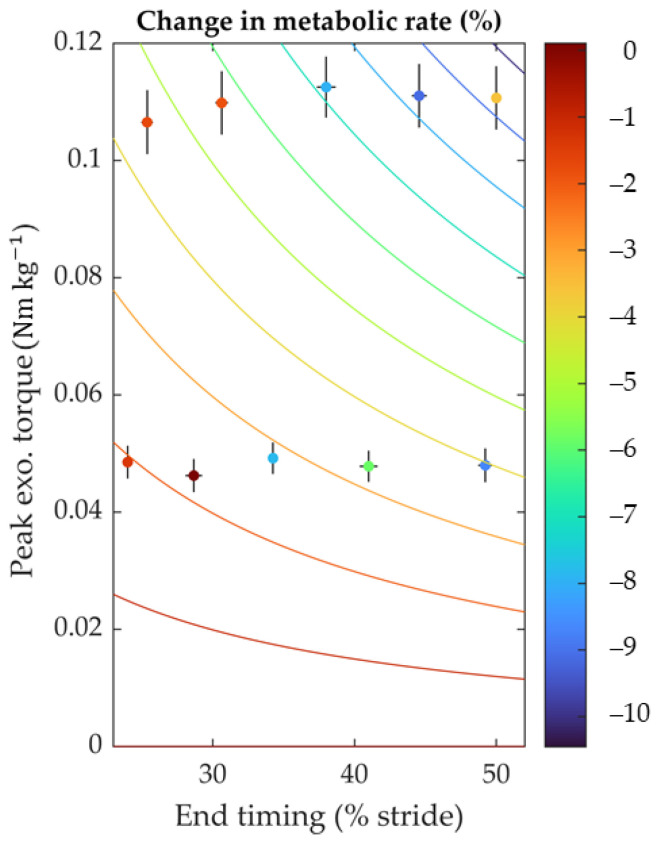
Effect of timing and magnitude on metabolic rate change. The colored dots represent ten conditions. A color scale characterizes the metabolic rate change versus the no-assist condition. End-timings are shown on the horizontal axis, and peak assistance magnitude is shown on the vertical axis. Colored contour lines visualize a trend from a linear mixed-effects model fitted to the data in Equation (2) (${\dot{\Delta E}}_{metabolic}$ ≈ −1.68 × *τ_exo_* × *t_exo_*). Error bars denote the standard error of the mean, illustrating the variation in actual timings and magnitudes across participants.

According to the following equation, we observed a significant effect of timing and magnitude on the change in metabolic rate versus the no-assist condition (Figure 5).

(3) $${\dot{\Delta E}}_{metabolic\;}\approx -1.68\times \tau_{exo}\times t_{exo}$$

In this equation, ${\dot{\Delta E}}_{metabolic}$ represents the change in metabolic rate, expressed in percentage, and *t_exo_* and *τ_exo_* represent the desired end-timing (% gait cycle) and peak torque magnitude (Nm kg^−1^). The interaction term p was the only one required to fit the data (*p*-value of interaction term: p_Mag×Tim_ = 9 × 10^−4^) significantly. The other terms from the initial model (Equation (2)) were insignificant and were thus removed during the stepwise elimination. The trend shows that conditions with greater peak torques and later end-timing produced a greater reduction in metabolic rate. The largest decrease in metabolic rate was 9.1 ± 12.5% (mean ± S.D., *p* = 0.047, paired *t*-test between Powered-On and Powered-Off conditions). This reduction occurred in the highest assistance magnitude and the second-latest end-timing. None of the conditions reduced the metabolic rate compared to the No-Exo condition. The metabolic rate in the No-Exo condition was 4.8% lower than in the best assistance condition.

We also observed significant interactions of timing and magnitude effects on the mean biological hip extension moment, preload work rate, and push-off work rate:

(4) $$\bar{\tau_{hip+}}\;\approx \;-0.011\times \tau_{exo}\times t_{exo}+0.361$$

(5) $${\dot{W}}_{Preload+}\;\approx \;0.001\times \tau_{exo}\times {t^{2}}_{exo}\;-\;1.759$$

(6) $${\dot{W}}_{Push-off+}\;\approx \;-0.114\times \tau_{exo}\times t_{exo}+3.470\times \tau_{exo}+1.873$$

**Figure 6:**
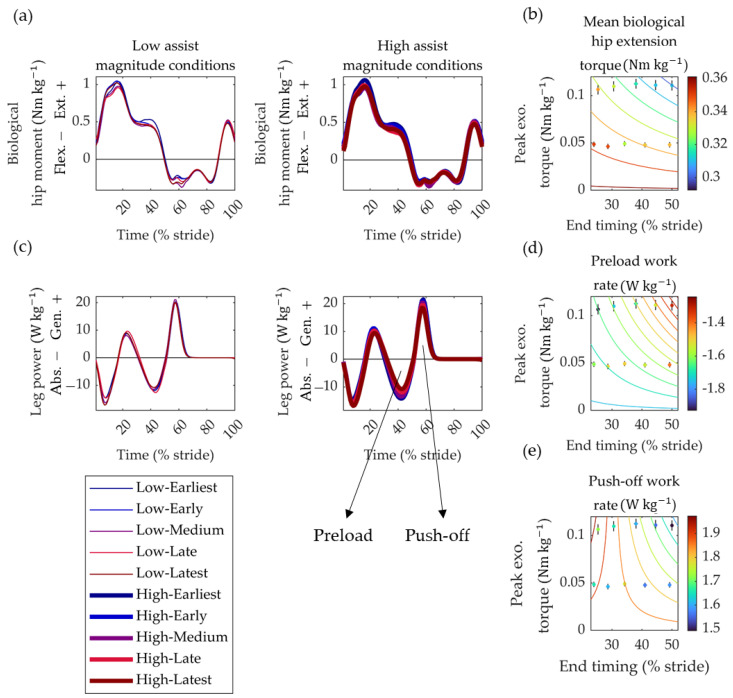
Effect of timing and magnitude on biomechanical parameters. (**a**) Biological hip moment. Positive values represent extension moments, and colored lines in the left figure represent the different timings with low assistance magnitude. Colored lines in the right figure represent different timings with high assistance magnitude. (**b**) Effect of timing and magnitude on the mean positive biological hip extension moment from panel a. (**c**) Leg power calculated using the individual limb method [39]. Arrows indicate the different work bursts showing significant effects in panels d and e. (**d**) Preload work rate from panel c. (**e**) Push-off work rate from panel c. Colored lines in panels d and e represent the trends from the linear mixed-effects models.

Whereby *t_exo_*, *τ_exo_*, and $\bar{\tau_{hip+}}$ represent the desired end-timing (% gait cycle), peak torque magnitude (Nm kg^−1^), and the mean of the biological hip extension moment (Nm kg^−1^). ${\dot{W}}_{Preload+}$ and ${\dot{W}}_{Push-off+}$ are the preload work rate and push-off work rate of the leg, respectively. All coefficients and intercepts in Equations (4)–(6) were statistically significant (all *p*-values were < 0.006). For each of these three biomechanical variables, the trends were such that later end-timings and greater assistance magnitude resulted in smaller peak torques and powers. No other significant effects were observed on ankle or knee torques and powers (Figure 6).

## 4. Discussion

The current study describes the design and evaluation of a semi-rigid hip exoskeleton. In the first part of the study, we explain how optimizing the proportional gain reduced the within-stride RMSE to about 1 Nm. In the second part, we describe how human experiments with the optimized exoskeleton show that, compared to the Powered-Off condition, the greatest reduction of 9.1% was found in the condition with the greatest peak torque and the second-latest end-timing. We found an interaction of magnitude but no isolated effects of timing or magnitude. Furthermore, none of the conditions reduced metabolic rate compared to the No-Exo condition.

We did not find a clear trend showing optimal stiffness or derivative gain’s effect with this exoskeleton. This contrasts with experiments involving other types of wearable robots, such as ankle exoskeletons or a robotic waist tether, which show the presence of an optimal series elastic stiffness that improves force tracking [29,31]. Regarding the investigation of the effect of stiffness, it is important to note that we used a range of coil springs with different dimensions and masses to cover the sweep range (McMaster-Carr, Elmhurst, IL, USA). These diverse material properties may affect the RMSE in various ways (for instance, springs with a heavier mass might oscillate differently). This variability could have contributed to the inability to observe a clear trend.

The achieved optimal RMSE was approximately 1 Nm, constituting around 35.5% of the peak torque in magnitude conditions and 15.2% in high-magnitude conditions. This substantial variability is likely attributed to the inherent challenges associated with a semi-rigid design, such as the movement of components on the participant. This variability may explain the relatively large fluctuations observed in metabolic effects. Our metabolic cost and biomechanical evaluation protocol identified a trend indicating a greater reduction with later end-timing and increased assistance magnitude. Other biomechanical parameters exhibiting significant trends concerning timing and magnitude help elucidate why later timing and magnitude contributed to a reduction in metabolic cost. As anticipated, we observed that the timing and magnitude that reduced metabolic cost also decreased the biological component of the hip extension moment. The greater reduction observed with increased magnitude and later end-timing is likely due to the exoskeleton torque more effectively covering a larger portion of the extension burst in the total hip moment.

More surprising were the reductions in leg power during the preload and push-off phases, where the push-off phase primarily relies on ankle push-off from the center-of-mass power. It is established that walking with a more intensive hip extension diminishes the need for push-offs and vice versa [42]. Hence, the hip exoskeleton’s greater magnitude and later-end timing may increase total hip extension and reduce the push-off requirement. The finding that later end-timing reduces metabolic rate aligns with results from studies on other hip assistance devices. Ding et al. used a human-in-the-loop approach to optimize peak-timing and end-timing, discovering that optimal conditions often featured an end-timing close to the maximal range set for the experiment [6]. In our study and theirs, the optimal end-timing was later than when the biological hip extension moment ended. This highlights that the optimal assistance pattern does not necessarily mirror biological kinetics [43]. 

Our study was carefully designed with a balanced recruitment of both men and women (four and six, respectively). While a power analysis was not initially conducted due to the unavailability of data specific to the research question, the sample size was planned before the study. The participants were not informed about the condition specifics or the randomization sequence, ensuring an unbiased response to the intervention. However, we acknowledge that the researchers were not blinded during the analysis. Conditions in our study were randomized to avoid any sequence effects that might influence the outcome measures. We included two reference conditions, Powered-Off and No-Exo, to evaluate the assistance conditions.

Our semi-rigid hip exoskeleton combines the advantages and disadvantages of rigid exoskeletons and soft exosuits. On the one hand, it has rigid components that allow greater force transfer than most exosuits. On the other hand, the absence of hinge joints allows greater freedom of movement. However, the rigid structure still hindered freedom of movement relatively more than soft exosuits, for example, by pressing into the soft tissue. Furthermore, the metabolic cost reductions achieved were less than the best performances in both categories: rigid exoskeletons and soft exosuits. While the semi-rigid form factor combines some advantages, the present result does not support the idea that semi-rigid is better.

Limitations of the study include the relatively high variability in the actual actuation patterns and their effects on metabolic cost. Due to this variability, no conditions were found to significantly reduce the metabolic rate compared to walking without the exoskeleton. In the No-Exo condition, the metabolic cost was approximately 4.8% lower than in the best assistance condition. Considering that the exoskeleton mass of 5.77 kg is primarily situated around the waist, and each additional kilogram results in roughly a 1% increase in metabolic cost [17], theoretically reducing the exoskeleton mass could bring the cost of the best assistance condition close to walking without the exoskeleton. Based on the literature review from Sawicki et al. [13], the maximum reduction in metabolic cost for the hip exoskeleton under walking conditions compared to walking without a device is 19.8% [10]. Recently, it was also possible to reduce metabolic cost with an entirely passive hip exosuit [44]. The results obtained with our present device do not approach these best-in-class performances since it increased metabolic cost compared to walking without an exoskeleton rather than reducing it. We recognized that the initial prototype of our exoskeleton was heavier than desired, and we applied conservative torque limits, considering it was the first iteration of our device. These factors contributed to our inability to demonstrate metabolic rate improvements compared to the No-Exo condition. In future research, we intend to refine the exoskeleton by reducing its weight and investigating the effects of higher torque. The exoskeleton could work better in populations with a more impaired gait; however, this is unknown at this point, and the exoskeleton could also be less effective in such populations. Exploring how the device performs with clinical populations, including patients with mobility issues, stroke survivors, and individuals with Parkinson’s disease, could provide valuable insights. Our current prototype was only tested during level ground walking at a constant speed. A study by Voloshina et al. [45] demonstrates how the body works and utilizes energy differently during walking on various terrains. For future improvements, conducting additional tests on surfaces such as inclines and uneven ground at different speeds would provide a better understanding of how our semi-rigid exoskeleton functions. This expanded testing could also enhance the practical application of exoskeletons in everyday life. Presently, we do not know how the exoskeleton would perform under different conditions, such as walking with added loads. Based on studies conducted by Mooney et al. [1,19], we anticipate that the percent change in metabolic cost would be slightly lower when walking with added load. Research from their group demonstrated an 11% reduction in metabolic cost during walking with an exoskeleton without added load [19] and an 8% reduction during walking with the same exoskeleton while carrying a 23 kg load [1]. To achieve greater reductions in metabolic cost, it will likely be necessary further to enhance the fit and comfort of the exoskeleton design and optimize other parameters of the actuation pattern (e.g., onset time, peak time, peak magnitude) as these parameters can impact metabolic cost [46]. Optimizing multiple parameters simultaneously could be achieved through human-in-the-loop optimization. Our group is developing lighter and simpler passive elastic exoskeletons to address some of the above limitations. 

## 5. Conclusions

The present study demonstrates the effects of a semi-rigid design in assisting walking, making progress compared to previous efforts with semi-rigid designs that predominantly focused on static activities, such as sit-to-stand transitions. Our findings revealed a trend of greater reductions in metabolic cost with later timings and greater magnitudes. Compared to walking with the exoskeleton powered off, the largest reduction, 9.1%, was observed with an end-timing at 44.6% of the stride cycle and a peak magnitude of 0.11 Nm kg^−1^. None of the tested conditions reduced the metabolic cost below that of walking without the exoskeleton. Further refinement of the exoskeleton design and optimization of other aspects of the actuation pattern will be necessary to reduce metabolic costs substantially.

## Data Availability

The data presented in this study are publicly available in Supplementary Data File S1.

## Supplementary Materials

The following supporting information can be downloaded at: https://www.mdpi.com/article/10.3390/biomimetics9040211/s1, Data File S1: MATLAB source data of Metabolic cost and Biomechanics evaluation protocol. Data File S2: Video showing walking with the bilateral semi-rigid exoskeleton.
